# Identifying and Tracking Pedestrians Based on Sensor Fusion and Motion Stability Predictions

**DOI:** 10.3390/s100908028

**Published:** 2010-08-27

**Authors:** Basam Musleh, Fernando García, Javier Otamendi, José Mª Armingol, Arturo de la Escalera

**Affiliations:** 1 Intelligent Systems Laboratory, Universidad Carlos III de Madrid/ Avda de la Universidad 30, 28911, Leganés, Madrid, Spain; E-Mails: bmusleh@ing.uc3m.es (B.M.); fegarcia@ing.uc3m.es (F.G.); armingol@ing.uc3m.es (J.M.A.); 2 Universidad Rey Juan Carlos/ Paseo Artilleros s/n. 28032, Vicálvaro, Madrid, Spain, E-Mail: franciscojavier.otamendi@urjc.es (J.O.)

**Keywords:** pedestrian detection, advanced driver assistance systems, stereo vision, laser technology, confidence intervals, sensor fusion

## Abstract

The lack of trustworthy sensors makes development of Advanced Driver Assistance System (ADAS) applications a tough task. It is necessary to develop intelligent systems by combining reliable sensors and real-time algorithms to send the proper, accurate messages to the drivers. In this article, an application to detect and predict the movement of pedestrians in order to prevent an imminent collision has been developed and tested under real conditions. The proposed application, first, accurately measures the position of obstacles using a two-sensor hybrid fusion approach: a stereo camera vision system and a laser scanner. Second, it correctly identifies pedestrians using intelligent algorithms based on polylines and pattern recognition related to leg positions (laser subsystem) and dense disparity maps and u-v disparity (vision subsystem). Third, it uses statistical validation gates and confidence regions to track the pedestrian within the detection zones of the sensors and predict their position in the upcoming frames. The intelligent sensor application has been experimentally tested with success while tracking pedestrians that cross and move in zigzag fashion in front of a vehicle.

## 1. Introduction

Trustworthy sensors are key elements regarding current road safety applications. In recent years, advances in information technologies have lead to more intelligent and complex applications which are able to deal with a large variety of situations. These new applications are known as ADAS (Advance Driver Assistance Systems). In order to provide reliable ADAS applications, one of the principal tasks involved is obstacle detection, especially for those obstacles that represent the most vulnerable road users: pedestrians. In terms of quantifying the accident rate depending on the type of transportation, pedestrians, who account for 41% of the total number of victims, represent the largest number of traffic accident victims in terms of deaths. It is well known that human errors are the cause of most traffic accidents. The two main errors are drivers’ inattention and wrong driving decisions. Governments are trying to reduce accidents with infrastructure improvements and educational campaigns, but they cannot be completely eliminated due to the human factor. That is why ADAS can reduce the number, danger and severity of traffic accidents. Several ADAS, which nowadays are being researched for Intelligent Vehicles, are based on Artificial Intelligence and Robotics technologies.

On-board perception systems are essential to estimate the degree of safety in a given situation and to allow the control system to make a suitable decision. Traffic safety research, developed around the world, shows that it is not possible to use only one sensor to get all relevant information from the road environment, making data fusion from different kinds of sensors necessary.

In this article a novel fusion method is proposed. The method combines the information provided by a 2D laser range finder and a stereo camera to detect pedestrians in urban environments. By combining both sensors, limitations inherent to each one can be overcome. Laser range sensors provide a reliable distance to the closest obstacles, thus giving trustable information of the surrounding, but this information is limited due to the low amount of data provided by the device and occlusions. With this lack of information, estimation of the type of obstacles found in a road environment is a tough task. On the other hand, data provided by computer vision systems have more information but less structured. This information can be very useful when trying o estimate the type of obstacle *i.e.*, pedestrian detection, but less precise to give a robust localization. A fusion system can be helpful to fulfill the requirements of such exigent applications as vehicle safety systems. It also can assure than in situations when one of the sensors is not available the other one can be used to allow the application to work under the hardest conditions

The objectives that are addressed are:

1. Identification of pedestrians and tracking of their trajectories. The focus is to detect the objects that are in the environment, classify the pedestrians and track them modeling their trajectory and identify possible collisions.
2. Installation of an intelligent system in the vehicle that tells the driver of potential dangers.

The tools that are going to be used are:

1. The sensors that allow for the acquisition of data from the environment.
2. Statistical inference or decision making to perform a probability calculation on the prediction of the trajectories.
3. Algorithms that will match the measurements and the predictions so as to classify the objects and determine their exact location, and send alarms in case the object is too close to the vehicle.

## 2. State of the Art

Statistics show that more than a half of accidents resulting in fatal victims happened in urban environments, in other words, where the active safety vehicle’s systems, e.g., ABS, ESP, have lower influence. Because of that, ADAS for front-side collisions, pedestrian run-over or automatic emergency braking are attracting an increasing interest. In addition, systems aiming to protect the most vulnerable users of these infrastructures such as pedestrians, cyclists, *etc.*, are difficult to develop due to the great variety of shapes, sizes and appearances involved [1].

Sensor data fusion [2] has been proposed in order to improve the performance, both in localization and robustness, of algorithms developed for detecting obstacles in urban environments. Making use of sensorial fusion techniques the perception of the environment can be improved as well as making up for the incompleteness of sensors which have partial faults or provide limited information. Current perception systems are designed based on multi-sensor design using computer vision (monocular or stereoscopic) in the visible spectrum and infrared and laser sensors, lidar or radar [3,4].

There are some constraints related to perception systems design that have to do with coverage, precision, uncertainty, *etc.* One of these problems is the limitation in spatial coverage. Usually a unique sensor is used to cover a reduced area; perhaps a higher coverage can be achieved doing data fusion from several sensors [5]. Limited temporal coverage is produced by the time needed to obtain and transmit a measurement by the sensor. Increasing the number of sensors used when making data fusion will reduce these limitations.

Another aspect to consider is the sensorial imprecision, inherent to the nature of sensors. Measurements obtained by each sensor are limited by the precision of the sensor used. The higher the number of sensors is, the higher the level of precision that is achieved in data fusion [6].

There is a new problem when designing perception systems to be applied to Intelligent Transportation Systems – uncertainty – which depends on the object observed instead of the sensor. It is produced when some special characteristics (such as occlusions) may appear when the sensor is not able to measure all attributes relevant to perception or when the observation is ambiguous [7]. A unique sensor may be unable to reduce the uncertainty in its perception due to its limited vision of the object [8]. This kind of situations comes up frequently in urban environments, where pedestrians, streetlights, etc, constantly appear blocked by parked vehicles, stopped in the street, *etc.*

Fusion methods are typically divided into two types according to the level in which fusion is performed: low level fusion, so called centralized fusion schemes [9], and high level fusion, so called decentralized schemes. Low level schemes perform fusion using a set of features extracted from both sensors. High level fusion performs different classifications with data provided by each sensor separately. A final stage combines information from all classifications. Each configuration has its own advantages and disadvantages [10].

Low level fusion combines information from both sensors creating a new set of data with more information, but problems related to data association arise. Low level approaches that take advantage of statistical knowledge [9,11] obtain information from all sensors and combine the information using Bayes formula, Support Vector Machines (SVM), Neural Networks, *etc.*

High level fusion schemes allow fusion in an easier and more scalable way; new sensors can be added more easily but with less information to do the classification. They can be differentiated in track based fusion and cell based fusion schemes. The first one tries to associate the different objects found in each sensor [12]. The second one [13] uses occupation grids, adding confidence according to the type of sensor that detects the obstacle, but losing the geometrical structure.

Other works related to fusion schemes take advantage of laser scanner trustworthiness to select regions of interest where vision-based systems try to detect pedestrians [6,14]. In [2] detection of especially dangerous zones is done using laser scanner information integrated along time. In [7], information from different sensors creating a feature vector is used to perform an unique classification (called medium level schemes).

## 3. The IvvI Project

**Figure 1.**
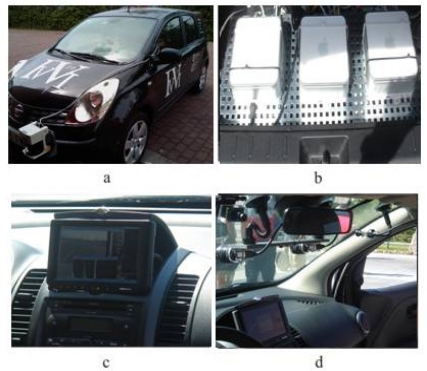
IvvI research platform.

IvvI (Intelligent Vehicle based on Visual Information, Figure 1a) is a research platform for the implementation of systems based on computer vision and laser technology, with the goal of developing ADASs. The purpose of the IvvI platform is to test perception algorithms under real conditions, and five sensing capabilities are being researched for Lane Keeping System, Adaptive Cruise Control, Pedestrian Detection, Traffic Sign Recognition and Driver Drowsiness Detection.

Research results are being currently implemented in a Nissan Note (Figure 1a). There is a DC/AC power converter connected to an auxiliary vehicle's battery. Through it, the electrical power needed for the computers, cameras and laser is obtained. There is a CMOS color camera for the detection of traffic signs and other vertical signs (Figure 1d) and another CMOS color camera inside the car to detect drowsiness situations. A binocular stereo vision system (Figure 1d) is used for pedestrian and lane detection during day driving, whereas an infrared camera placed on the wing mirror is used for pedestrian detection during night driving. A laser placed on the front bumper is used for pedestrian and vehicle detection (Figure 1a). There are three PCs (Figure 1b) in the vehicle's boot which are used for processing the information captured by the sensors.

The laser and the stereo vision system are the input sensors to the fusion based tracking algorithm, which is the main line of research of this article. The algorithm provides information about the environment to the driver. This is done through a monitor (Figure 1c), similar to a GPS device that many vehicles carry nowadays, where the pedestrians are graphically represented and, above all, through the vehicle loudspeakers that play several warning messages depending on the location of the pedestrian and the seriousness of the possible run over.

## 4. General Description of the Algorithm

**Figure 2.**
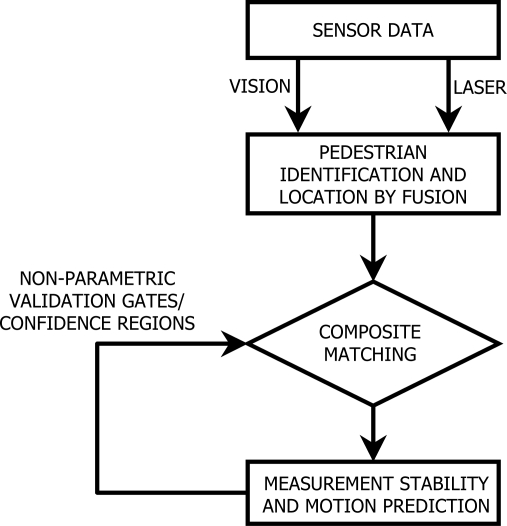
Tracking framework.

The intelligent tracking algorithm (Figure 2) looks for the correct classification of objects as well as for their exact location. Its main step is the matching of the data or measurements obtained by the different fused sensors and the predictions on the tracked location of the pedestrian. These predictions are based on continuously monitoring the stability of the sensor measurements for the very near past, that is, for the last set of frames and for different time increments, so that changes in directions and speeds are accounted for.

The individual-sensor data is to be jointly fused by the proper calibration and coordination algorithms. It is necessary that each and every sensor perform a measurement exactly at the same moment in time so that a composite fused measurement might be obtained. The fusion step has to account for an absence of measurements by any or all of the devices, so that the trajectories and the stability of the time movements are statistically tracked. Raw data is recorded at time t in multiple dimensions [in this case, two (x_t_, y_t_)]; then data is converted into movements that the pedestrian has performed in a time increment l (*Δ^1^* *x_t_*, *Δ^l^* *y_t_*) so they are used as the basis for the predictions.

Two sets of statistical inference procedures are to be performed. The first procedure is the analysis of the stability of the displacements, that is, the analysis of the consistency or the homogeneity of the current measurement with the previous movements of the same time increment. The stability hypothesis is usually tested using confidence intervals or validation gates in one dimension and simultaneous confidence regions in multiple dimensions [15,16].

The second procedure is the prediction of the motion of the pedestrian or his/her location at a particular future time. Based on the current location and the stable movements for different time increments, it is possible to set confidence regions for the location at future times t+l [17]. These predictions are to be made for each pedestrian independently.

The matching algorithm confronts then the fused stable measurements for different time increments with all the location predictions that have been made in previous moments of time. If within the validation gates, that is, with the occurrence of proper multiple matches, the known pedestrians are liable to be continuously tracked. If no match is achieved, new pedestrians may be available for tracking.

After each successful classification or tracking stage, the predictions must be updated, because changes in directions or velocity may very likely occur. By performing moving predictions, that is, taking into account only measurements for past short time intervals, these changes will not negatively affect the predictions and ruin the tracking of the proper trajectories.

What follows is a detailed explanation of each of the stages of the algorithm. Section 5 explains the laser subsystem including its detection and classification stages. Section 6 details the computer vision system and its pedestrian identification step. Section 7 is then used to address the intelligent tracking algorithm, which is tested in Section 8 with real experiments in an urban outdoor environment. Section 9 is finally used to present the conclusions and future work.

## 5. Laser Subsystem

The aim of the laser subsystems is to detect pedestrians based on the data received from the laser scanner device. The laser, a SICK LMS 291-S05, has a measurement range of 80 meters and a frequency up to 19 frames per second. The detection process is composed of two stages. In the first stage, the data is received and obstacles’ shapes are estimated. A second stage performs obstacle classification according to the previously estimated shape. In the present research, pedestrian classification is performed by searching through specific patterns related to leg positions.

### 5.1. Obstacle segmentation

**Figure 3.**
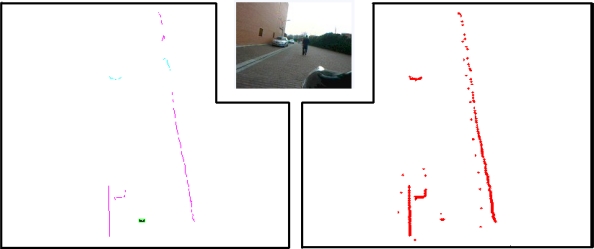
Environment information given by the algorithm after obstacle segmentation. **Left:** shape estimation using polylines; detected pedestrian is highlighted. **Center**: Real image captured by a camera mounted in the vehicle. **Right**: raw data captured by the laser after egomotion correction.

The laser scanner provides a fixed amount of points that represents the distance to the obstacles for a given angle, from the coordinate origin situated in the bumper of the vehicle. This measurement is taken from a single laser that performs a 180° rotation. Thus, there is a time difference between each distance measured. Due to the vehicle movement and laser scanner rotation there is a variation along time included in the measures, therefore vehicle egomotion correction is mandatory before processing the data; this is done thanks to an on-board GPS-IMU system. The resulting points are joined according to the Euclidean distance among them (Figure 3).

After the clustering algorithm, polylines are created, which join the points contained within segments. These polylines give information about shape and distance of the obstacle to the vehicle.

### 5.2. Pedestrian classification

**Figure 4.**
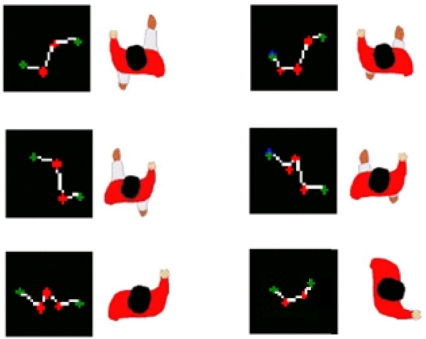
Pattern given by a pedestrian, according to leg situation. This pattern may appear rotated.

Classification is performed according to obstacles’ shape [18]. A study was performed to observe the pedestrian pattern during the walking process. Specific patterns were searched to identify a single pedestrian only using the information provided by the laser radar. Observations showed the movements patterns described in Figure 4.

Observation showed that most of the patterns shared a common feature, consisting of two different 90 degrees angles. This pattern was checked under different conditions and movements including test for standing pedestrians facing the laser and lateral standing pedestrians. Regarding to lateral standing pedestrians test showed that the pattern given by the laser includes the two mentioned angles by getting the whole shape of a leg. Taking advantage of such behavior a static model was created.

**Figure 5.**
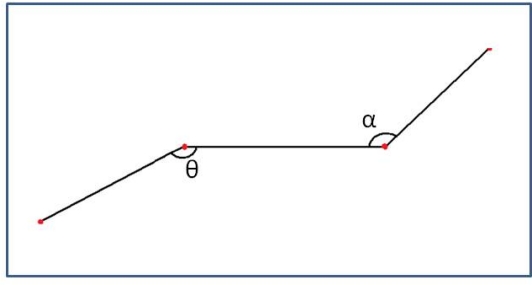
Model for pedestrian where the two angles of interest are detailed.

The process followed to match the found pattern, including rotation, consists on a first segmentation according to obstacle’s size and a final matching based on polylines’ shape. Segmentation computes the size of the polyline and checks that the detected obstacle has a size proportional to a human being. An obstacle that fulfills the size requirements is marked as candidate to be a pedestrian. An additional stage compares it with the model. The comparison stage links every two consecutive angles (Figure 5) with polylines and gives a similarity percentage according to equations (6) to (8):

(6) $$S_{\theta }=1-\frac{\frac{\pi }{2}-\theta }{\frac{\pi }{2}},$$

(7) $$S_{\alpha }=1-\frac{\frac{\pi }{2}-\alpha }{\frac{\pi }{2}}$$

(8) $$S=S_{\alpha }\cdot S_{\theta }$$

A single similarity score is computed for each of the two angles separately by comparing their value with the ideal $\frac{\pi }{2}$ [equations (6) and (7)]. Then, the total aggregated value, which is calculated by multiplying both single scores, measures the similarity of the measurements to the model.

If the case arises where more than three polylines are present, the algorithm is applied to every pair of consecutive angles and those with the highest values are chosen as the polyline similarity value. A threshold is used to classify the obstacle as a pedestrian.

## 6. Vision Subsystem

The purpose of this subsystem is also to detect and classify pedestrians; the detection range is 30 meters and a frequency up to 10 frames per second. In order to have depth information in computer vision it is necessary to set two cameras: in the IvvI is a stereo Bumblebee camera by Pointgrey. This system automatically performs the necessary rectification step [19–21].

Once the two images are correctly rectified, our proposed algorithm develops the dense disparity map and “u-v disparity” [22] to perform the analysis of the environment and the pedestrians. These tasks have got a high computational cost; therefore NVIDIA CUDA framework [23,24] is used to process in the GPU (Graphics Processing Unit).

### 6.1. Dense disparity map

The disparity map represents the depth W of every image point. The depth is calculated as follows:

(9) $$W=f\cdot B/(u_{L}-u_{R})=f\cdot B/d$$

where d is the disparity, f is the focal length and B is the baseline distance. (u_R_,v_R_) and (u_L_,v_L_) are the projection in the camera planes for the right and left cameras respectively of the point P = (U,V,W)^T^ of the world.

For this calculation to be valid, the two image planes must be coplanar, their optical axes must be parallel and their intrinsic parameters must be the same. It is therefore necessary to find the correspondence between points of the right and left images to determinate the disparity d (known as the stereo matching problem), using the following rectification:

(10) $$v_{L}/f=V/Z;v_{R}/f=V/Z\Rightarrow v_{L}=v_{R}$$

**Figure 6.**
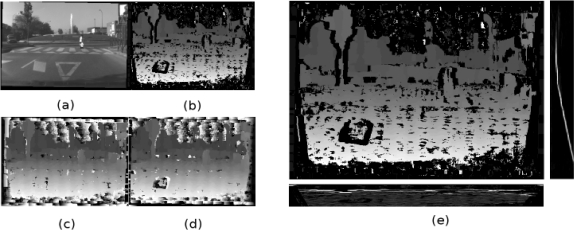
**(a)** Left image. **(b)** Cross-Checking image. **(c)** Left disparity map image. **(d)** Right disparity map image. **(e)** Disparity map and its corresponding v-disparity on the right and u-disparity below.

There are several possible solutions to this stereo matching problem in order to obtain the dense disparity map. Our algorithm follows the taxonomy presented by Scharstein and Szeliski in [25], where they propose that stereo algorithms are performed by the following four steps:

- Matching cost computation: Although there are more accurate cost functions [26], squared differences (SD) is preferred because it is faster and easier to implement in GPU processing. SD assumes equal gain in both cameras; that is why both images are pre-processed by Laplacian of Gaussian (LoG).
- Cost (support) aggregation: There are different kinds of support regions, and their choice influences in the resulting disparity map [27]. The algorithm implemented is based on square-windows support regions for cost aggregation because it is better in relation to GPU performance and the resulting disparity map is accurate enough.
- Disparity computation: There are mainly two methods for disparity computation: local [25] and global algorithms [28]. The local method WTA (Winner-take-all) is chosen. For a posterior disparity refinement task, the disparity map for the left image (left disparity map) and for the right one (right disparity map) are constructed. To avoid redundant computations, it is possible to use computations from the left disparity map to construct the right disparity map.
- Disparity refinement: This step tries to reduce the possible errors in the disparity map, which are usually produced in areas where texture does not exist, in areas near depth discontinuity boundaries [29], or in areas where there are repeated patterns, for example, on walls of buildings. For instance, enough texture does not exist either in the sky or in the road for images of driving environments, as figure 6a shows. The errors in the disparity map are likely to appear in these areas, see Figures 6c and 6d. To reduce these possible errors, a cross-check is performed; the result is shown in Figure 6b.

### 6.2. U-V disparity

Once the disparity map has been generated, it is possible to obtain the “u-v disparity”. As there is a univocal relationship between disparity and distance, the v-disparity expresses the histogram over the disparity values for every image row (v coordinate), while the u-disparity does the same but for every column (u coordinate). In short, the u-disparity is built by accumulating the pixels of each column with the same (u, d) and the v-disparity by accumulating the pixels of each row which the same (v, d). An example is illustrated in Figure 6e.

If it is assumed that obstacles have planar surfaces, every one appears in the u-disparity image as pixels whose intensity is the height of that obstacle. As the u-disparity image dimensions are the width of the original image and the height is the disparity range, those pixels have the same horizontal coordinate than the obstacle in the disparity map and the vertical coordinate is the disparity value of the obstacle. Regarding v-disparity, as its image dimensions are the disparity range and the height of the original image, the obstacles appear as vertical lines in its corresponding disparity value [30] as they are at the same disparity or distance. Another interesting feature is that the ground appears as an oblique line. This feature is very useful because the pitch, θ and height, h, of the cameras can be measured for each frame [31]. This information will be used to determine accurately the obstacle localization in the world coordinates.

### 6.3. Obstacle detection

**Figure 7.**
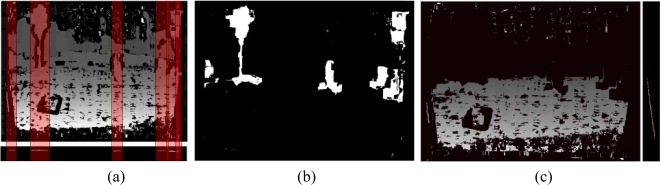
**(a)** Disparity map and thresholded u-disparity with the thresholding areas. **(b)** Thresholded disparity map where appear the obstacles in the study region. **(c)** Disparity map for a study region without obstacles and the road profile as red line obtained by means of the Hough transform.

The main goal of this system is to determine the regions of interest (ROI), which will be later on used to conclude if the obstacles are pedestrians or not. In order to do that, the road profile is estimated by means of the v-disparity [31]. This is why planar road geometry is assumed, which is reasonable at close areas in front of the vehicle. There are other obstacles detection systems which use the u-v disparity, such as the proposed in [32,33]. Our obstacle system is divided into the following three steps:

1. The first step is a preliminary detection over u-disparity. This task consists in thresholding the u-disparity image to detect obstacles which have a height greater than a threshold. This way the “thresholded u-disparity” is constructed at the bottom of Figure 7a. Blobs analysis is made on the binary image to determine the total number of obstacles and their horizontal position and width.
2. In the disparity image, the subimages defined by the horizontal obstacle position and width, red squares in Figure 7a, are thresholded using the disparity ranges obtained before, Figure 7b. They are the obstacles in front of the vehicle. This binary image is used as a mask to obtain a disparity map without obstacles and a partial v-disparity is constructed, where the road profile is extracted as a line, corresponding to equation (11), by means of the Hough transform (see Figure 7c):
  (11) v=m⋅d+b
  The pixels of the obstacles are eliminated because in the event of appearing numerous obstacles, the profile road may be incorrect [34]. The equation (11) represents the road profile as a function of the v image coordinate, the disparity d and the constant b and the road slope m.
3. Finally, a second blob analysis is performed to determine obstacles features, area and position, on the thresholded disparity map, Figure 7b. On the basis of this features, regions of interest are constructed on the visible left image for a posterior processing.

### 6.4. Obstacles localization

The obstacles’ localization in world coordinates (U, V) can be obtained, and it is a function of the image coordinates (u, v) of the contact point between the obstacles and the ground. In order to do that, equations (13) is obtained from equations (9) (11) and (12), where the parameter Cu corresponds to u coordinate of the optical center and θ is the pitch of the stereo rig. In this way, the obstacles localization is computed with more resolution than if the disparity values are used exclusively.

(12) $$U=\frac{(u-\mathrm{Cu})V}{f}$$

(13) $$\begin{array}{l} W=\frac{\mathrm{fBm}}{v-b}\text{cos}(\theta )\\ U=\frac{\left(u-\mathrm{Cu})B\cdot m\cdot \text{cos}(\theta \right)}{v-b} \end{array}$$

### 6.5. Obstacle classification

**Figure 8.**
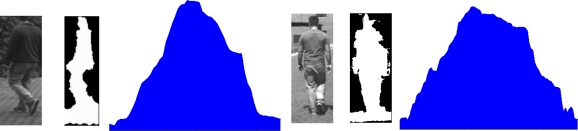
Two examples of pedestrians, their silhouette and their vertical projection.

The classification divides the obstacles into two groups: pedestrians and non-pedestrians. The result of the classification algorithm is a confidence score for the fact that the obstacle is a pedestrian; it is compared with a threshold and if it is greater, the obstacle is classified as a pedestrian. This classification is based on the similarity between the vertical projection of the silhouette and the histogram of a normal distribution. Figure 8 illustrates two examples of the vertical projection of a pedestrian silhouette from two different viewpoints, where both vertical projections are similar to the histogram of the normal distribution. The vertical projection for each obstacle is computed by means of the ROIs in the thresholded disparity map, which are results of the obstacles detection algorithms.

In order to characterize the vertical projection, the standard deviation, σ, is computed as if the vertical projection was the histogram of a normal distribution. In order not to make the standard deviation be a function of the obstacle dimension or independent on the obstacle localization, the standard deviation is divided by the width of the ROI getting σ_w_. This standard deviation will be used to compute the score.

**Figure 9.**
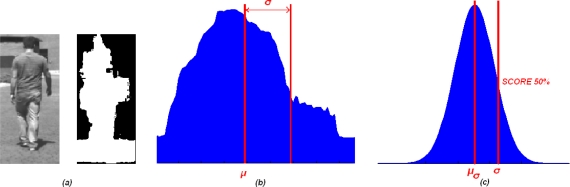
Process scheme to obtain a pedestrian score. **(a)** Pedestrian image and his silhouette. **(b)** The vertical projection of the pedestrian silhouette. **(c)** Normal distribution of the standard deviations and the score for the σ corresponding with the vertical projection of the pedestrian silhouette.

Several vertical projections of pedestrian have been processed to obtain their standard deviations; these standard deviations follow a normal distribution N(μ_σw_,σ_σw_). In order to compute the score for an obstacle, its standard deviation is used to obtain the value of the probability density function, where the maximum score 100% is produced if the standard deviation is equal to μ_σw_ and gets worse when the standard deviation is different from μ_σw_ (Figure 9).

## 7. Measurement Stability and Motion Prediction

Once the sensors and their corresponding algorithms have taken measurements individually and processed them in order to identify and classify objects as pedestrians, it is necessary to provide the intelligent system with tools that track the pedestrians and alert the driver to possible imminent collisions. In this section statistical models are developed to robustly infer the possible routes based on the current position as well as near past locations.

### 7.1. Background on errors in multiple statistical inference

Inference is the part of statistics that relates sample information with probability theory in order to estimate or predict the value of one or several unknown parameters or compare two hypotheses about their value. The hypotheses are called null (which is the one statistically proven to be true or false according to a pre-specified confidence level γ) and alternative (which is chosen whenever the null is rejected).

In individual hypothesis testing about a single parameter φ, an observed value x is compared against a threshold value that result of the application of the confidence level γ, and a decision is taken by deciding to reject or not reject (accept) the null hypothesis.

It is well known, however, that two errors can occur when a decision of this kind is made about the value of one parameter: the null hypothesis is rejected when it should have been accepted (false positive or false detection, significance level = ω =1 − γ) or accepted when it should have been rejected (false negative or false standard, probability = β, testing power = 1− β). Table 1 depicts this decision-making problem.

In multiple testing (Table 2), the number of tests is large (M), as many as parameters, and the process should distinguish between null hypotheses which are really true (O) and those which are really false (A).

If ω is used in each individual test, the probability of “false positive” errors increases considerably: the probability of accepting only and all null hypotheses when they are true is only (1−ω)^O^. Global confidence is reduced, and is therefore not 1−ω but 1−Ω, where Ω is the global level of significance, much higher (worse) than the theoretically desired ω. On the other hand, when a large number of null hypotheses are rejected by this procedure, the error of failing to discover relevant alternative hypotheses is practically zero. In other words, as the relevant hypotheses are overestimated, practically all non-rejected null hypotheses are really standard. This approach therefore favours the determination of all significant and some other hypotheses (false positives, which could be numerous) as relevant, in exchange for having no false negatives.

Therefore, in order to make a correct decision for an aggregate level of significance Ω, and prevent too many false positives from occurring, the form in which individual testing is performed has to be adjusted. Individual tests have traditionally been maintained, although the level ω has been adjusted. Usually, ω is adjusted and controlled in two different ways.

The first is known as the Bonferroni correction [16]. The level of significance of each test is individually reduced from ω to ω/M, so the p-values must be much lower for a null hypothesis to be individually rejected. As the number of significantly relevant tests is reduced, the number of false positives also diminishes. The number of false negatives, however, or the number of null hypotheses which should have been rejected, increases considerably. The correction, therefore, by attempting to avoid “false positives”, gives rise to too many “false negative” errors.

The same occurs with the Sidak adjustment [35], in which ω is reduced to:

(14) $$\omega =1-{\left(1-\Omega \right)}^{1/M},$$

maintaining the global level of significance Ω. If M is high and Ω is low, ω will be very close to zero and it will be difficult to reject (by very small p-values) any individual null hypotheses, and no false null hypotheses will be rejected. These two traditional corrections, then, favour the determination of all non-significant or standard and some significant (false negatives) hypotheses as relevant.

### 7.2. Background on robust confidence intervals on correlated parameters

Robust multivariate hypothesis testing involves the simultaneous comparison of sample values with thresholds. One possible way to set the thresholds is to use confidence intervals, whose main purpose is the estimation of the value of one or several unknown parameter. Confidence intervals come in the form of confidence limits or prediction thresholds. If a new sample value lies within the confidence limits, the value is said to be homogeneous with the previous values, accepting the null hypothesis of belonging to the same underlying distribution. Otherwise, the value is said to belong to a different distribution, rejecting the null and accepting the alternative.

Confidence intervals (CI) on a single unknown parameter are a means to set thresholds on its values and have the form φ∈[ φ_−_, φ_+_] or CI_φ_ or alternatively φ∈ [φ^*^-k_1_ (V(φ^*^))^1/2^, φ^*^+k_2_ (V(φ^*^)^1/2^)], where φ* is the point estimator of the parameter and V(φ*) the variance of the estimator.

The estimation problem relates therefore to the proper identification of the distribution of the control statistic and the associated probability calculation based on the confidence level that results in the k_i_ values. If the distribution is not known, an upper threshold on the value of the k_i_ might be however readily calculated using Chebishev’s inequality, $k=k_{1}=k_{2}=\sqrt{\frac{1}{\omega }}$, with the corresponding non-parametric confidence interval on one parameter being $\varphi \in [\varphi^{*}-\sqrt{\frac{1}{\omega }}{\left(V(\varphi^{*})\right)}^{1/2},\varphi^{*}+\sqrt{\frac{1}{\omega }}{\left(V(\varphi^{*})\right)}^{1/2}]$.

However, in most real situations, there are more than one variable or parameter involved in the decision making or inference process. In other words, confidence intervals are to be set for several parameters, which are usually correlated.

The first possibility is to set individual and independent confidence intervals for each and every variable, but adjusting the confidence percentage to account for the multivariate situation according to Bonferroni’s or Sidak’s corrections, as well as the setting bounds using Chebishev’s inequality. The corresponding confidence region comes in the form of a rectangle in two dimensions, a prism is three dimensions and a polyhedron in more dimensions [36].

**Figure 10.**
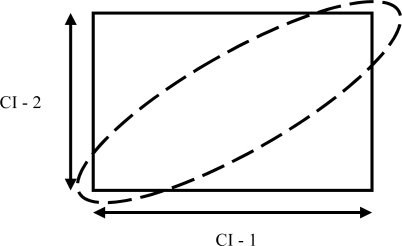
Confidence regions in two dimensions.

However, it has been shown that if the variables are correlated, the false negative rate is very high, since the response area covered by the combination of individual CI’s is much larger than what it should be. The corresponding area should come in the form of an ellipse in two dimensions and an ellipsoid in higher dimensions. Figure 10 explains the problem in two dimensions.

The equation of the ellipsoid, or the ellipse in two dimensions, in terms of the Mahalanobis standardized distances of each point to the center of the ellipse, E, is as follows [36]:

(15) $$\frac{{\left(\frac{x-\bar{x}}{s_{x}}\right)}^{2}+{\left(\frac{y-\bar{y}}{s_{y}}\right)}^{2}-2R\left(\frac{x-\bar{x}}{s_{x}}\right)\left(\frac{y-\bar{y}}{s_{y}}\right)}{{\left(1-R\right)}^{2}}=D=E^{2\text{max}},$$

where the constant D is the multivariate equivalent to the value k in one dimension, that is, the maximum distance measured in standard deviations from the center of the confidence region to the fringe of the ellipse. D is approximated as D = k * 7/1.5 [37].

### 7.3. Application to the tracking of pedestrians

Multivariate statistical models are ready to be particularized in this article to the movement of pedestrians. The data is measured at each time t individually from the sensors: (x_s,t,i_; y_s,t,i_) where s = 1, …, S sensors and s = f for the fused values with as many measurements as objects i = 1, … I are detected.

The values in absolute units are also transformed into movements or displacements Δ^l^x_s,t,i_ and Δ^l^y_s,t,i_, where l = 1,…L accounts for the time interval used to calculate the displacements:

$$\begin{array}{l} \Delta^{l}x_{s,t,I}=x_{s,t,I}-x_{s,t-1,I}\;\forall s,\;\text{i},1\\ \Delta^{l}y_{s,t,i}=y_{s,t,I}-y_{s,t-1,I}\;\forall s,\;\text{i},1 \end{array}$$

The first set of models relate to the control of the stability of the displacements. For each object i, the last C values are used to calculate the averages on the moves $\bar{\Delta^{l}x_{s,t,i}},\bar{\Delta^{l}y_{s,t,i}}\;\forall \;s,\;\text{i},1$, as well as the standard deviations *Δ^l^* *sx_s,t,i_* *Δ^l^* *sy_s,t,i_* ∀ s, i, l, and the correlation among dimensions *Δ^l^* *R_s,t,i_*.

The confidence intervals might readily be calculated using Chebishev’s inequality and Sidak’s corrections:

(16) $$\begin{array}{l} \Delta^{l}x_{s,t,i}\in [\Delta^{l}x{-}_{s,t,i},\Delta^{l}x{+}_{s,t,i}]=\bar{\Delta^{l}x_{s,t,i}}\pm \sqrt{\frac{1}{1-{\left(1-\Omega \right)}^{\left(1/M\right)}}}*\Delta^{l}sx_{s,t,i}\;\forall s,\;\text{i},\text{ l}\\ \Delta^{l}y_{s,t,i}\in [\Delta^{l}y{-}_{s,t,i},\Delta^{l}y{+}_{s,t,i}]=\bar{\Delta^{l}y_{s,t,i}}\pm \sqrt{\frac{1}{1-{\left(1-\Omega \right)}^{\left(1/M\right)}}}*\Delta^{l}{\mathrm{sy}}_{s,t,i}\;\forall s,\;\text{i},\text{ l} \end{array}$$

Similarly, the confidence regions or ellipsoids (*Δ^l^* *x_s,t,i_* ; *Δ^l^* *y_s,t,i_*), Ellipses,t,i, are:

(17) $$\begin{array}{l} \frac{{\left(\frac{\Delta^{l}x_{s,t,i}-\bar{\Delta^{l}x_{s,t,i}}}{\Delta^{l}{\mathrm{sx}}_{s,t,i}}\right)}^{2}+{\left(\frac{\Delta^{l}y_{s,t,i}-\bar{\Delta^{l}y_{s,t,i}}}{\Delta^{l}{\mathrm{sy}}_{s,t,i}}\right)}^{2}-2\left(\Delta^{l}R_{s,t,i}\right)\left(\frac{\Delta^{l}x_{s,t,i}-\bar{\Delta^{l}x_{s,t,i}}}{\Delta^{l}{\mathrm{sx}}_{s,t,i}}\right)\left(\frac{\Delta^{l}y_{s,t,i}-\bar{\Delta^{l}y_{s,t,i}}}{\Delta^{l}{\mathrm{sy}}_{s,t,i}}\right)}{{\left(1-\Delta^{l}R_{s,t,i}\right)}^{2}}=D^{2}\\ \forall \;s,i,l \end{array}$$

The second set of models are used to determine where the object is going to be at t + l, by just adding the average observed move to the current position:

(18) $$\begin{array}{l} {}^{l}x{-}_{s,t,i}=\Delta^{l}x_{s,t,i}-\left(\bar{\Delta^{l}x_{s,t,i}}-\sqrt{\frac{1}{1-{\left(1-\Omega \right)}^{\left(1/M\right)}}}*\Delta^{l}{\mathrm{sx}}_{s,t,i}\right)\\ {}^{l}x{+}_{s,t,i}=\Delta^{l}x_{s,t,i}+\left(\bar{\Delta^{l}x_{s,t,i}}+\sqrt{\frac{1}{1-{\left(1-\Omega \right)}^{\left(1/M\right)}}}*\Delta^{l}{\mathrm{sx}}_{s,t,i}\right)\\ {}^{l}y{-}_{s,t,i}=\Delta^{l}y_{s,t,i}-\left(\bar{\Delta^{l}y_{s,t,i}}-\sqrt{\frac{1}{1-{\left(1-\Omega \right)}^{\left(1/M\right)}}}*\Delta^{l}{\mathrm{sy}}_{s,t,i}\right)\\ {}^{l}y{+}_{s,t,i}=\Delta^{l}y_{s,t,i}+\left(\bar{\Delta^{l}y_{s,t,i}}+\sqrt{\frac{1}{1-{\left(1-\Omega \right)}^{\left(1/M\right)}}}*\Delta^{l}{\mathrm{sy}}_{s,t,i}\right)\\ \left(lx_{s,t,i}{;}^{l}y_{s,t,i}\right)\in \;{\text{Ellipse}}_{s,t,i}^{*}\;\forall \;s,\;\text{i},\;\text{l}\;\text{and}\;\text{s}=f \end{array}$$

The total number of prediction models, M, is M = (S + 1) * I * L * 3.

### 7.4. Composite matching and classification

The last stage in this multivariate assessment is the location of the pedestrians. After the discussion in the previous sections, the information available at each time t is:

- The measurements from the sensors.
- The confidence bounds or validation gates for the prediction of moves for each object and lag, for each sensor and dimension as well as for the fused data, and in combined confidence regions
- The validation gates for the prediction of location for each object and lag, for each sensor and dimension as well as for the fused data, and in combined confidence regions. The algorithm then must confront the raw data, the lagged data and the fused data with the validation gates and prediction regions so as to assign the measurements to an existing or new object. There exist several possible results:
  - All the validation gates and confidence regions are positively met for one of the existing objects. The measurement is assigned to that object, which continues to be a pedestrian or another object, fixed or not.
  - None of the validation gates or confidence regions are met. A new object is created and starts to be tracked.
  - If either the gates for the stability of moves or the position gates are met, due to a no-read or a sudden change in direction or velocity, the measurement is assigned to same object which continues to be tracked.

## 8. Experimental Results

**Figure 11.**
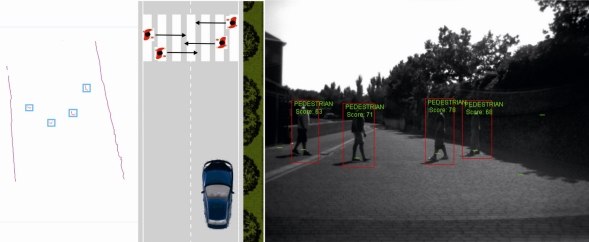
Pedestrian detection by the IvvI vehicle.

The following experiments have been carried with the IvvI vehicle outdoors in order to evaluate the robustness and reliability of the proposed detection and tracking algorithm. Figure 11 shows the capability of the perception system to detect multiple objects and identify them as pedestrians. The figure shows four pedestrians crossing in front of the vehicle, two in each direction. The vision image shows boxes around the identified pedestrians. The laser frame shows possible pedestrians surrounded by boxes after processing the raw data.

The data obtained out of the sensorial system has been used to test the performance of the fusion algorithm under different real conditions: crossings of pedestrians while moving in zigzag and changes of speeds.

### 8.1. Pedestrians crossing and changing directions

The IvvI vehicle is first set on the road to test the proposed intelligent fusion-based tracking system outdoors, where pedestrians wander following both linear and non-linear paths.

#### 8.1.1. Definition of the experiment

**Figure 12.**
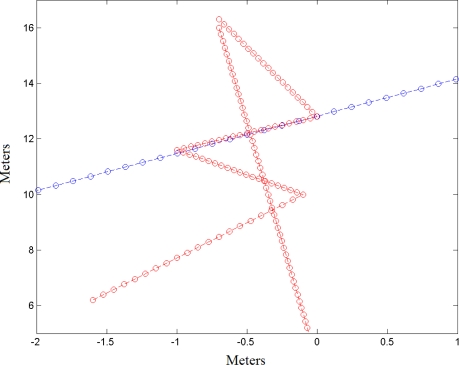
Paths followed by pedestrians.

Two pedestrians move for 29.2 seconds (292 frames) in front of the vehicle following the paths included in Figure 12. The trajectories are highlighted by the crossing of the two pedestrians and a single pedestrian changing direction in a zig-zag fashion.

#### 8.1.2. Parameterization of the tracking algorithm

The parameter S, the number of sensors, is set to S = 2, as a camera and a laser are used to obtain data from the environment. The parameter I, the count of objects, is set to I = 2, as two are the pedestrians being tracked. The parameter L, the number of time intervals, is set to L = 3 to allow for a quick execution of the algorithm. The parameter M, or the number of simultaneous tests that are performed at each t is M = (S + 1) * I * L * 3 = 54. The parameter C, or the number of past data used to calculate the trajectories and the moves, is set to C = 10, since that is the value corresponding to the number of maximum frame rate of the camera. The parameter Ω, or the overall significance level, is set to 5%.

Therefore: $k=\sqrt{\frac{1}{1-{\left(1-\Omega \right)}^{\left(1/M\right)}}}=\sqrt{\frac{1}{1-{\left(1-0.05\right)}^{\left(1/54\right)}}}=32.45≅33$

D = k * 7/1.5 = 119.89 ≅ 120.

#### 8.1.3. Analysis of the crossing

**Figure 13.**
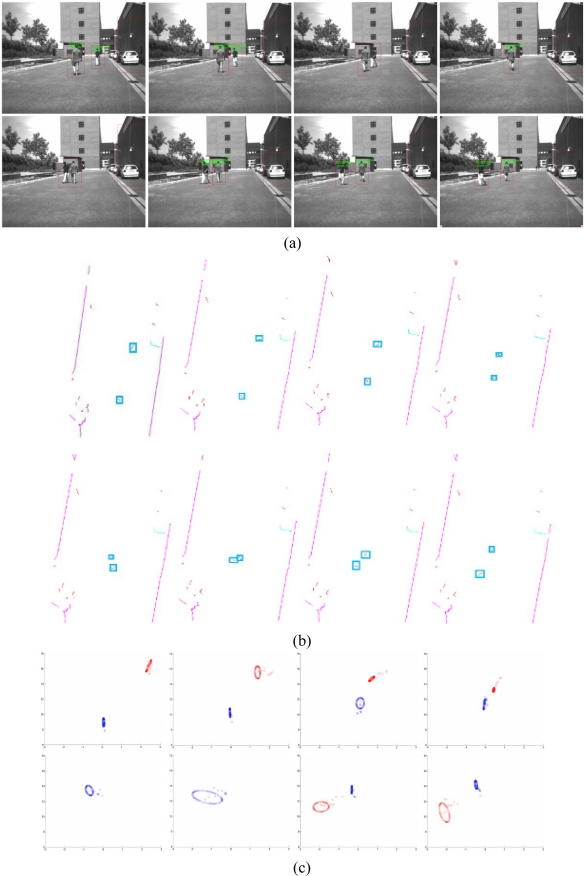
**(a)** Sequence of the crossing resulting from the vision subsystem. **(b)** Sequence of the crossing resulting from the laser subsystem. **(c)** Sequence of the crossing resulting from the tracking algorithm.

The cross happens in between frame number 70 to 90, or 2 second. The information provided by the two sensor systems, as well as the result of the tracking algorithm are included in Figure 13.

The tracking images show the ellipses corresponding to a time interval of 1 frame. At the time of the crossing, the algorithm is only able to classify one pedestrian. The result is a larger prediction region that covers both pedestrians. It also allows for the tracking of both as depicted in the figures corresponding to frames 95 and 100.

#### 8.1.4. Analysis of the Zigzag movement

**Figure 14.**
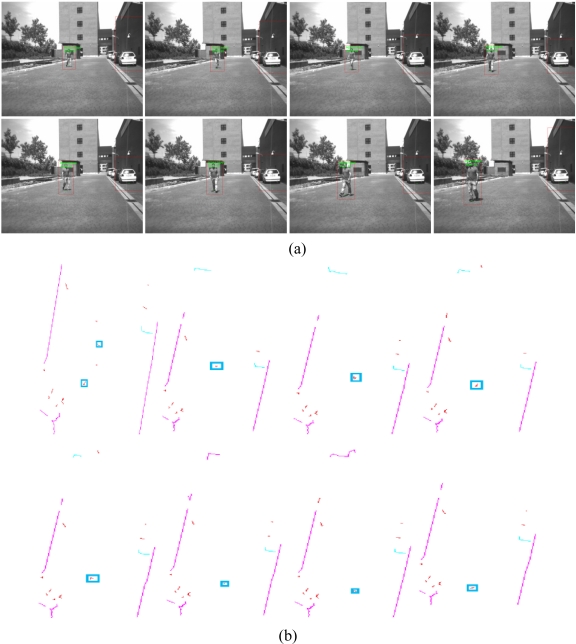

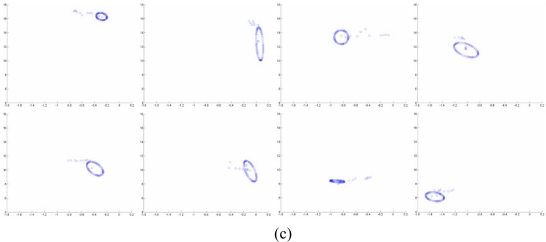
**(a)** Images sequence of a pedestrian with zigzag trajectory resulting of the vision subsystem. **(b)** Images sequence of a pedestrian with zigzag trajectory resulting of the laser subsystem.

The pedestrian changes directions between frames 205 and 270 for more than 6 seconds (Figure 14). The changes are properly picked but with the penalty of carrying larger ellipses, due to the increase in the value of the calculated standard deviations.

#### 8.1.5. Reliability results

Table 3 shows the absolute frequency distribution of measurements by each of the sensors (V = vision, L = laser, F = fusion) for each of the two pedestrians as well as the false positives. The first pedestrian should be detected in all of the 292 frames, whereas the second one only for the first 192 frames. The camera shows an additional still object for 288 of the 292 frames and the laser shows also the presence of another still object. By fusing the sensors, the 2 pedestrians are clearly picked in 257 frames, with the other 35 frames picking just one of the two. The number of false positives reaches 3, since in one frame 5 objects are picked other than the two still objects.

The hit rate per pedestrian (Table 4) is above 86% for the fused data. The hit rate is calculated as the percentage of reads over the count of frames in which the pedestrians were correctly situated in front of the vehicle.

If C consecutive reads were not available, then it is not possible to calculate neither an average of the data nor a filtered value, and thus, the hit rate diminishes.

Table 5 includes the null acceptance ratio or the percentage of time in which the new value falls within the all the validation gates and confidence regions, both individually and jointly for the stability of the movements and the tracking of the trajectories.

The percentage is over 85% and should increase in uncontrolled environments whenever the variability of the measures is higher. In controlled environments, the pedestrian knows that it is being tracked and acts consistently so that the calculated standard deviation is smaller than it is when variability is speed and direction is more likely to occur. In fact, the hit rate is higher for the first pedestrian who at one point follows a zigzag pattern.

### 8.2. Changes in speeds of both pedestrian and vehicle

**Figure 15.**
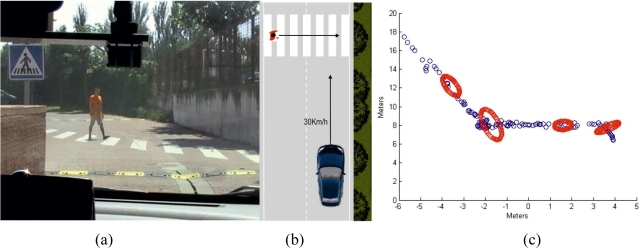
**(a)** Visualization from the vehicle. **(b)** Zenithal map of the situation. **(c)** Sequence of the crossing resulting from the tracking algorithm.

An additional experiment is performed to assess the performance of the algorithm when the pedestrian is changing speeds while the vehicle is moving (Figures 15a and 15b).

The vehicle starts detecting the pedestrian about 18 meters before the zebra crossing (Figure 15c). The vehicle keeps on approaching the pedestrian up until a distance of 8 meters, as shown by the portion the graph that has a negative slope. Then, both the pedestrian and the vehicle stop. When the pedestrian sees that the vehicle has come to a full stop, he starts walking again at a higher pace. The vehicle does not start moving until the pedestrian has completely crossed the road, about three metres to the right of the vehicle; the graphs during these frames shows no slope. The vehicle starts its movement again, with the graph presenting a negative slope. The graph shows as well the prediction ellipses every 50 frames.

## 9. Conclusions and Future Work

Sensors are ubiquitous in today’s world, although it is necessary to give them with autonomy to process the information they get from the environment. Our research aims at developing intelligent sensors in a demanding field like Intelligent Transportation Systems. More specifically this article addresses the problem of the identifying and classifying objects and pedestrian so as to help drivers to avoid accidents.

Developments in both hardware and software are necessary to create robust and intelligent application in Advanced Driver Assistance Systems. The sensorial fusion of a laser and a computer vision system as well as a classification algorithm has proven successful for the tracking of pedestrians that cross and wander in zigzag in front of the vehicle.

Original algorithms have been developed for classification and tracking. A new approach to pedestrian detections based on a laser and variable models has been presented, giving an estimation of how close they are to the ideal pattern for a pedestrian. Regarding the stereo-vision subsystems two original contributions are worth mentioning. First, the implementation of the disparity map construction with the cross-checking and the u-v disparity using CUDA in order to obtain a real time system. Second, a novel and fast procedure for pedestrian identification using the silhouette of the stereo image has been presented. The success of the matching procedure is based on the application of non parametric multivariate statistics to the localization problem while tracking pedestrians. More specifically, the Sidak correction has been applied to calculate the proper multivariate significance level, the Chebishev inequality has been used to account for non-normality and confidence regions have been calculated to determine the positioning of the pedestrians in the upcoming frames. Two other contributions have made for the robustness of the algorithm. The use of movements and not raw measurements has allowed for the proper control and dimensioning of the confidence regions. The check for stability of the measurements prior to the calculation of the predictions has also increased the hit ratio while recognizing and classifying pedestrians. All experiments have been performed in real environments using the IvvI research platform, where all the algorithms have been implemented and tested.

Improvements should be made on the perception as well as the tracking systems to improve the hit rate. The classification of the obstacles detected by the stereo system can have more features into account. Once the obstacles have been detected and their size and distanced to vehicle found, methods that use several image features like [38] can be applied and still work in real-time. Experiments with more pedestrians are also currently being carried out. Future works will be focused on other kind of obstacle detections such as cyclist or motorists. Visual information has already been used in some works to detect them. Laser Scanner detection algorithm is being developed for these obstacles by adding new information to the models such as movement, width, *etc.* Now, the system warns the driver if there is a crossing of trajectories, but in [39] several decision mechanisms have been implemented that evaluate behavioral alternatives based on sensory information and internal prediction. This way the system would decided when the best behavior is a warning message to the driver or taking control of the vehicle to avoid a pedestrian or to minimized the severity of the injures.

## Figures and Tables

**Table 1.** Test-related decision-making problem.

		**Decision made**	
		**Accept null**	**Reject null**
**Null hypothesis**	**True**	CORRECT DECISION	ω - False positive or relevance
	**False**	β - False negative or standard	CORRECT DECISION

**Table 2.** Decision-making problem in multiple testing’s.

	**ACCEPTED H_0,m_**	**REJECTED H_0,m_**	**TOTAL**
**TRUE NULLS**	*P*	*F* – False detections	*O*
**FALSE NULLS**	*N* – False acceptance	*T*	*A*
**TOTAL**	*W*	*R*	*M*

**Table 3.** Distribution of detections per frame.

	**MEASUREMENTS**									
	TOTAL	292	292	292	192	192	192	292	292	292
		**PEDESTRIAN 1**			**PEDESTRIAN 2**			**OTHER**		
		**V**	**L**	**F**	**V**	**L**	**F**	**V**	**L**	**F**
**DETECTIONS**	0	66	131	34	31	148	27	0	36	0
	1	226	161	125	161	44	125	288	172	35
	2			133			40	4	68	171
	3								15	69
	4								1	16
	5									1

**Table 4.** Hit rates per pedestrian.

	**HIT RATE (%)**	
	PEDESTRIAN 1	PEDESTRIAN 2
VISION	77.40 %	85.64 %
LASER	56.51 %	23.40 %
FUSION	88.36 %	87.77 %

**Table 5.** Hit rate per model.

STABILITY	95.83 %	95.03 %
TRACKING	93.51 %	86.16 %
AT LEAST ONE	98.46 %	96.18 %
BOTH	90.98 %	85.28 %
